# Interface Rotation in Accumulative Rolling Bonding (ARB) Cu/Nb Nanolaminates Under Constrained and Unconstrained Loading Conditions as Revealed by In Situ Micromechanical Testing

**DOI:** 10.3390/nano15191528

**Published:** 2025-10-07

**Authors:** Rahul Sahay, Ihor Radchenko, Pavithra Ananthasubramanian, Christian Harito, Fabien Briffod, Koki Yasuda, Takayuki Shiraiwa, Mark Jhon, Rachel Speaks, Derrick Speaks, Kangjae Lee, Manabu Enoki, Nagarajan Raghavan, Arief Suriadi Budiman

**Affiliations:** 1 Xtreme Mechanics Laboratory, Engineering Product Development (EPD), Singapore University of Technology and Design (SUTD), 8 Somapah Road, Singapore 487372, Singapore; 2 Nano-Micro Reliability Laboratory (MRL), Engineering Product Development, Singapore University of Technology and Design (SUTD), Singapore 487372, Singapore; 3 Center for Advancing Materials Performance from the Nanoscale (CAMP-Nano), State Key Laboratory for Mechanical Behavior of Materials, Xi’an Jiaotong University, Xi’an 710049, China; 4 Industrial Engineering Department, BINUS Graduate Program—Master of Industrial Engineering, Bina Nusantara University, Jakarta 11480, Indonesia; 5 Research Center for Structural Materials, National Institute for Materials Science, 1-2-1 Sengen, Tsukuba 305-0047, Ibaraki, Japan; 6 Department of Materials Engineering, School of Engineering, The University of Tokyo, 7-3-1 Hongo, Bunkyo-ku, Tokyo 113-8656, Japanenoki@rme.mm.t.u-tokyo.ac.jp (M.E.); 7 Institute of High Performance Computing (IHPC), Agency for Science, Technology and Research (A*STAR), 1 Fusionopolis Way, #16-16 Connexis, Singapore 138632, Singapore; 8 Department of Manufacturing and Mechanical Engineering and Technology (MMET), Oregon Institute of Technology, Klamath Falls, OR 97601, USA; 9 Oregon Renewable Energy Center (OREC), Oregon Institute of Technology, Klamath Falls, OR 97601, USA

**Keywords:** multilayers, nanolaminates, interface-based plasticity mechanism, nanoplasticity

## Abstract

Accumulative rolling bonding (ARB) Cu/Nb nanolaminates have been widely observed to exhibit unique and large numbers of interface-based plasticity mechanisms, and these have been associated with the many extraordinary properties of the material system, especially resistances in extreme engineering environments (mechanical/pressure, thermal, irradiation, etc.) and ability to self-heal defects (microstructural, as well as radiation-induced). Recently, anisotropy in the interface shearing mechanisms in the material system has been observed and much discussed. The Cu/Nb nanolaminates appear to shear on the interface planes to a much larger extent in the transverse direction (TD) than in the rolling direction (RD). Related to that, in this present study we observe interface rotation in Cu/Nb ARB nanolaminates under constrained and unconstrained loading conditions. Although the primary driving force for interface shearing was expected only in the RD, additional shearing in the TD was observed. This is significant as it represents an interface rotation, while there was no external rotational driving force. First, we observed interface rotation in in situ rectangular micropillar compression experiments, where the interface is simply sheared in one particular direction only, i.e., in the RD. This is rather unexpected as, in rectangular micropillar compression, there is no possibility of extra shearing or driving force in the perpendicular direction due to the loading conditions. This motivated us to subsequently perform in situ microbeam bending experiments (microbeam with a pre-made notch) to verify if similar interface rotation could also be observed in other loading modes. In the beam bending mode, the notch area was primarily under tensile stress in the direction of the beam longitudinal axis, with interfacial shear also in the same direction. Hence, we expect interface shearing only in that direction. We then found that interface rotation was also evident and repeatable under certain circumstances, such as under an offset loading. As this behaviour was consistently observed under two distinct loading modes, we propose that it is an intrinsic characteristic of Cu/Nb interfaces (or FCC/BCC interfaces with specific orientation relationships). This interface rotation represents another interface-based or interface-mediated plasticity mechanism at the nanoscale with important potential implications especially for design of metallic thin films with extreme stretchability and other emerging applications.

## 1. Introduction

Bimetallic nanolaminates have been experimentally analyzed, simulated, and mathematically modeled to fulfil applications for strong/tough, wear-resistant, fracture-resistant, impact-resistant, and advanced structural materials [1,2]. A large number of interfaces in nanolaminates have the potential to modify bulk/local interfacial plasticity and thereby its strength and toughness as well as fracture/impact resistance [3,4]. Here, the test material is ARB Cu/Nb nanolaminate, which is being extensively considered for its remarkable flow strength, deformability, thermal stability, and radiation shielding due to its unique semi-coherent immiscible interface [5,6,7]. Cu/Nb nanolaminate has lattice mismatch which provides the capability to stop, absorb, and annihilate dislocation at its interface to mitigate strain localization under applied loading [8,9]. Shao et al. [10] noted that the existence of residual compressive stresses in Nb and residual tensile stresses in Cu may make dislocations bow in opposite directions in Cu/Nb nanolaminates. Zhang et al. [11] further highlighted the role of the interface in the plastic behavior of Cu/Nb nanolaminates under shock compression testing mode. They noted that critical shock pressures for nucleating and transmitting dislocations through a flat interface are considerably higher than those from a faceted interface owing to interface characteristics causing significantly diverse mechanisms for dislocation nucleation, absorption, and transmission.

Furthermore, the Cu/Nb ARB nanolaminate displays crystallographic anisotropy owing to the distinct interfaces along the rolling direction (RD) and the transverse direction (TD) [12,13]. Molecular dynamics simulations predicted that the Cu/Nb ARB nanolaminate’s interface is highly anisotropic and theoretically has highly prohibitive interfacial shear in the rolling direction and has a finite interface shear strength ~1.2 GPa in the transverse direction (TD) [14]. Budiman et al. [15,16] highlighted the role of interfaces in hindering crack propagation and affecting plasticity mechanisms. Radchenko et al. [16] further emphasize the impact of interface shear strength on fracture behavior, with differences observed amongst the transverse and rolling directions. Along the transverse direction (TD) of Cu/Nb ARB nanolaminate, Budiman et al. [15,16,17,18] noted notch widening during cross-layer fracture, indicating a significant extent of interfacial sliding/shearing attributed to size effects and localized shearing/plasticity mechanisms on the interfaces. Radchenko et al. [16] documented improved plasticity with low interface shear strength along the TD compared to the RD.

Local plastic instability due to strain localization has been observed experimentally during Cu/Nb nanolaminate micropillar compression. Zheng et al. [19] showed that, within the shear band formed during micropillar compression, the layers in Cu/Nb nanolaminate could rotate to orientations favorable for interfacial sliding. This phenomenon has also been observed in Cu/Nb ARB nanolaminates, indicating the significance of interfaces in the enhancement the ductility of such nanolaminated materials [12]. Furthermore, Demkowicz et al. [14] predicted the possibility of rotation of interfaces through molecular dynamic simulations during micropillar compression tests.

As the occurrence and propensity of interfacial plasticity along the TD is well observed [14,15,20,21], the intention of the current study is to document interfacial shearing along the RD in Cu/Nb ARB nanolayered samples, as well as to report if additional interfacial plasticity mechanisms such as rotation are evident and under which circumstances. Once the conditions under which interface shear/rotation along the RD are well documented, the insight could support the designer/researcher in their quest for designing structures or components of Cu/Nb nanolaminated materials workable under varying loading configurations/conditions. To achieve the above outcome, two sets of experiments were designed: (1) in situ rectangular micropillar compression essentially to achieve unconstrained interfacial shear/rotation and study its impact on interface-based localized shearing/plasticity mechanisms along the RD and (2) in situ microbeam bending tests of pre-notched Cu/Nb (63 nm) ARB clamped beams to examine constrained localized interfacial plasticity along the TD and interfacial rotation along the RD under offset loading. In the latter study, offset loading was introduced to examine the effect of local bending moment/bending load on localized interfacial plasticity (shear/rotation). The study documents Cu/Nb nanolaminate’s localized interface-based plasticity mechanisms during in situ rectangular micropillar compression and in situ microbeam bending, essential for interface engineering to enhance the ductility and achieve extreme stretchability of nanolayered composites. These outcomes of the study could be very significant for advanced structural applications, as they offer understandings of applied loading configurations/conditions and their effect on the interfacial shear/rotation along the TD and RD for anisotropic Cu/Nb nanolaminate. The authors think that information regarding interfacial sliding/rotation under diverse loading configurations could help researchers in scheming workable design diagrams for a given material system, which could motivate the design/fabrication of novel strong/tough assemblies sustainable under complex loading configurations/conditions for evolving functionalities, like stretchable bimetallic conductors for innovative wearable devices.

## 2. Materials and Methods

### 2.1. Assembly of ARB Cu/Nb Nanolaminates

Cu/Nb nanolaminates are created through accumulative roll bonding where alternating layers of pre-treated Cu and Nb are stacked and later rolled together. The rolled layers are then trimmed and stacked again and rolled repeatedly in the same rolling direction till the distinct layer thickness is shortened down to nanometers (63 nm for the test material in the work) [20]. During the ARB, the plastic deformation is performed along the ND. As the volume of the sample is conserved, the sample suffers expansion (rolling reduction of 99.999%) in the RD and no deformation along the TD. Therefore, the ARB produces different textures along rolling and transverse directions and thereby distinct interfacial characteristics [8,21,22,23,24]. Additional specifics of the accumulative roll bonding and the Cu/Nb texture development can be found in the literature [2,25].

### 2.2. Fabrication of Cu/Nb ARB Rectangular Micropillar Samples

**Figure 1:**
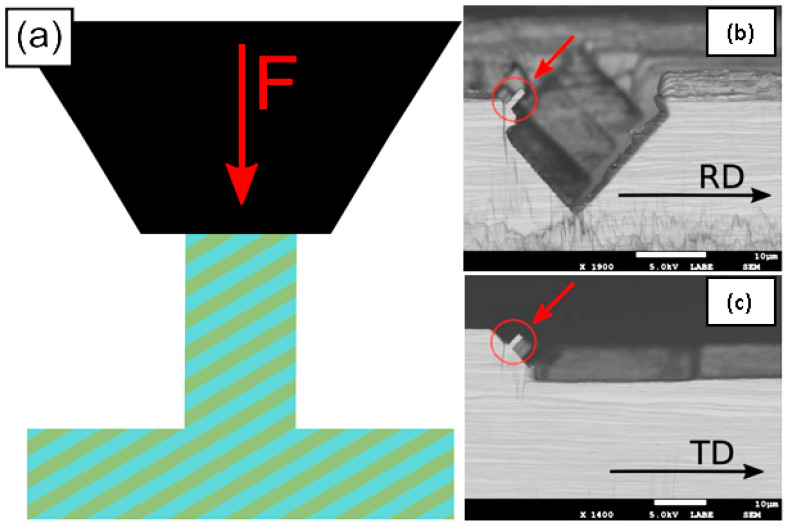
(**a**) Schematic of the in situ rectangular pillar compression experiments. SEM images of as-fabricated pillars with ±45° offset from (**b**) RD and (**c**) TD, respectively.

The Cu/Nb ARB nanolaminate rectangular micropillars for in situ pillar compression experiments were fabricated using FIB milling (FEI Nova Nanolab dual-beam FIB, Hillsboro, OR, USA) at Nanyang Technological University’s Microelectronics Reliability & Characterization Laboratory in Singapore. The milling process applied for the fabrication of micropillars consists of three milling stages, (a) a constant 30 kV acceleration voltage with 21 nA beam current for the first two stages of the milling and (b) in the third stage, a constant 30 kV acceleration voltage was applied, whereas the beam current was gradually reduced to 28 pA from 21 nA for polishing the sample. Two pillars were made with interfaces inclined 45° with regard to the loading axis. In this study, micropillars with rectangular cross-sections were used in contrast to the more typical circular pillars reported thus far in the literature [4,26,27,28,29,30]. The rectangular cross-section allows the direct testing of interface shear strength along the RD as well as the TD of the Cu/Nb ARB nanolaminate, as illustrated in Figure 1a and shown in Figure 1b,c. The width to height ratios (1:2 and 1:3) were selected to suppress buckling instability during pillar compression, as suggested by Zhang et al. [31]. Similar aspect ratios (1:2 and 1:3) were also used by Li et al. in their interface shear study of Cu/Nb PVD nanolaminates [32].

### 2.3. Fabrication of Cu/Nb ARB Microbeam Samples

**Figure 2:**
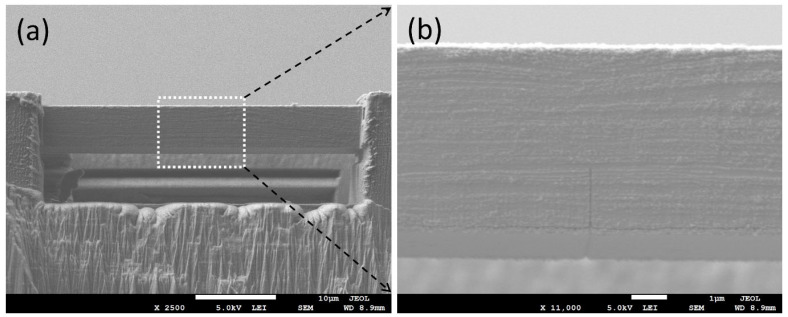
SEM images depicting front view of Cu/Nb (TD) Beam, (**a**) the full-frontal view of the beam with dimensions: L~40 µm, W~5 µm, and T~5 µm, (**b**) close-up of the frontal notch in the beam fabricated by FIB with dimensions: H = 1240 nm, W = 120 nm.

In order to fabricate Cu/Nb ARB microbeam samples, FEI Nova Nanolab dual-beam FIB at Nanyang Technological University’s (NTU) Microelectronics Reliability & Characterization Laboratory, Singapore was employed to manufacture clamped microbeams. FIB was used to prepare microbeams from pre-polished nanolaminates with ~63 nm layer thickness (see Figure 2). Initially, Cu/Nb ARB was milled at 30 kV with a high beam current to produce a clamped microbeam configuration. Subsequently, a low milling current was used for the finer cutting and polishing to the final dimensions. The center notches were milled at 10 pA current as a single line cut across the width of the beam. The notch depths were kept between 1/5 and 1/3 of the beam heights, whereas the notch widths ranged between 100 nm and 360 nm. Figure 2 shows representative images of the fabricated clamped microbeam. The microbeam dimensions were selected to satisfy both small-scale yielding and plane strain [33]. The small gradient height (height/thickness) typically permits stable crack expansion, whereas the large gradient height may prevent edge cracking for the above clamped microbeam configuration.

### 2.4. In Situ Microbeam Bending

The in situ microbeam bending tests were performed by means of a PI-85 SEM pico-indenter (Bruker^®^, Billerica, MA, USA) in JEOL FESEM (JSM-7600F), Tokyo, Japan. The microbeams were loaded by a truncated cone-shaped indenter with a top surface with diameter 5 µm. The bending load was applied using a displacement control mode through a fixed 5 nms^−1^ displacement rate. The generated load–displacement data was corrected for thermal drift, which was estimated to be of the order of ~10 µN. Two sets of experiments were performed: (a) microbeam bending with no offset loading (Cu/Nb-NOL), where the indenter tip is aligned along the notch of the microbeam and (b) microbeam bending with offset loading (Cu/Nb-OL), where the indenter tip loads the sample with an offset from the center of the notch along the length of the beam to generate a combination of local bending moment at the notch tip and bending load at the notch of the microbeam. The load–displacement data were thus generated and are discussed in the subsequent section below.

For both in situ micropillar compression and in situ microbeam bending tests, the load–displacement curve and corresponding real-time video were synchronized and taken using a frame grabber with the TriboScan software version 9 (for TI-950) (Bruker^®^, USA). Also, before the experiments, drift correction measurements were performed to then correct the final load–displacement measurements.

## 3. Experimental Results

### 3.1. In Situ Rectangular Micropillar Compression

The crystallographic anisotropy in Cu/Nb nanolaminates leads to different interfaces along the RD and TD [12,13]. Typically, in situ rectangular micropillar compression experiments provide a well-defined stress state, making them suitable for studying anisotropy effects [34]. Additionally, apart from mechanical anisotropy, in situ micropillar compression could also provide more direct insights into mechanical behavior earlier and/or later, surpassing the elastic limit until rupture [35]. Furthermore, the response of the material during micropillar compression tests can be quantitatively mapped to understand the stress and strain distribution at small length scales [36].

In the current work on Cu/Nb ARB nanolaminate samples, in situ rectangular micropillar compression experiments were performed along the RD as well as in TD to probe the interfacial plasticity mechanism. Molecular dynamics simulations have predicted that the Cu/Nb ARB interface is highly anisotropic and theoretically does not shear in the RD and has a finite interface shear strength of ~1.2 GPa in the TD [14].

**Figure 3:**
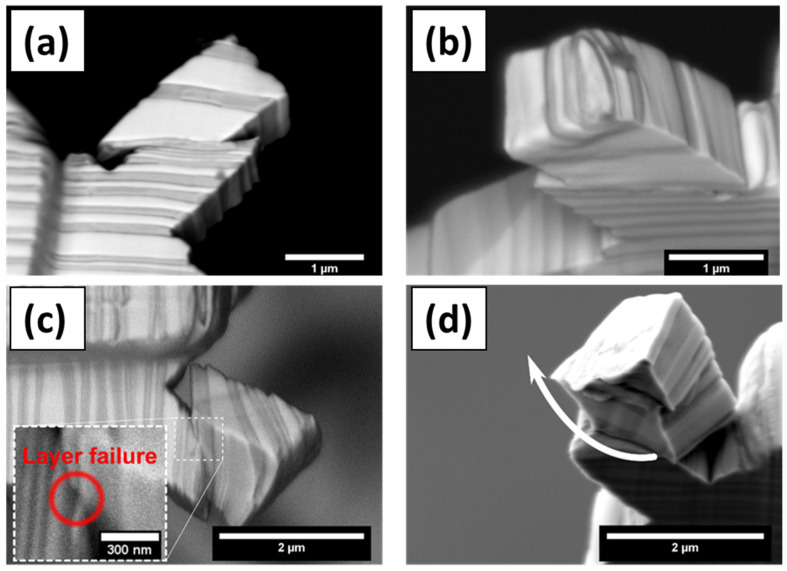
SEM images of the deformed TD (**a**,**b**) and RD (**c**,**d**) rectangular micropillars. The TD micropillar exhibits the interface shear along two interfaces of a single Cu layer, as can be seen from the back-scattered electron image in (**a**). The shear does not result in free Nb surface creation. Only the Cu layer surface is exposed, as can be seen from (**b**). In the case of the RD micropillar, the top part of the RD micropillar is rotated relative to its bottom part, as shown in (**d**). One layer near the rotation region is broken, as shown in the inset in (**c**). The micropillar was sheared in RD, and due to extreme stress concentration built up at one of the corners of the pillar, the shearing in the RD was resisted, and then the whole pillar was stuck in that corner, thus activating deformation of materials in the other direction (i.e., TD), thus the interface rotates.

**Figure 4:**
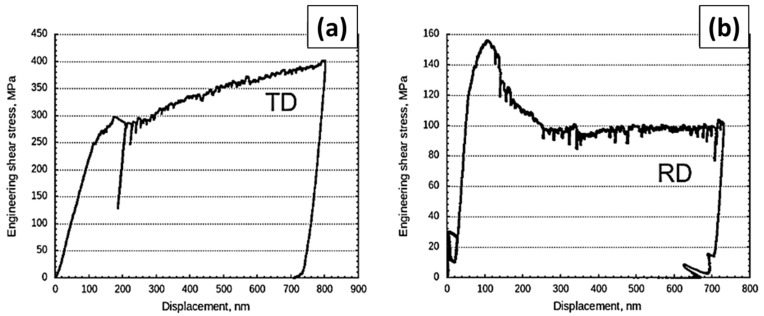
Engineering-resolved shear stress along the interface vs. indenter displacement for the TD and RD rectangular micropillars as shown in Figure 3: (**a**) The interface shear in the TD pillar is activated at ≈300 MPa shear stress followed by stress increase with deformation. The stress is underestimated here due to the decrease in the contact area upon pillar deformation and (**b**) the interfacial rotation (evident from Figure 3c,d) in the RD pillar occurs at ≈155 MPa immediately followed by decrease in stress.

The results of in situ rectangular micropillar compression experiments along the TD and RD are shown in Figure 3. Their corresponding engineering-resolved shear stress vs. displacement plots (see Figure 4). The engineering-resolved shear stress vs. displacement curves were attained from the experimentally obtained load vs. displacement curves using $\sigma_{eng}=-F\left(h_{0}-∆\right)/{A_{0}h}_{0}$ engineering-resolved shear stress if the shear direction follows the RD or TD according to the interface inclination, where *σ_eng_* is engineering stress, *F* is the measured load, ∆ is the measured displacement, *A*_0_ is an initial cross-section area of the pillar, and *h*_0_ is the initial pillar height.

#### 3.1.1. Interface Shear in the TD Rectangular Micropillar Compression

The ARB 63 nm Cu/Nb TD micropillar exhibits interfacial shear, which starts to occur at ≈300 MPa (see Figure 4a), which is much smaller than the 1000–1200 MPa predicted via MD simulations [14]. This may be attributed to its finite interface in the experiment compared to infinite interface modeled in the simulation. It is observed that dislocations moving out from a finite interface would encounter fewer misfit dislocations compared to an infinite interface modeled through MD simulation. The shear was only observed at one side of the interface, where a free Cu surface was formed after the shear (see Figure 3a,b). The inhomogeneity of the interface shear implies that the interface shear strength depends on the boundary conditions, and it is lower when a Cu free surface is formed and higher when a Nb free surface is formed.

The interface shear observed at two of the interfaces is initiated at ≈300 MPa and the stress keeps increasing with further deformation (see Figure 3a). Each load drop in the load vs. displacement curves corresponds to dislocation nucleation at the interface trailed by the dislocations leaving the interface [37,38]. The plasticity in the Cu layer is governed by the CLS and leads to plastic hardening. This plastic hardening is likely to be attributed to the observed increase in the stress (see Figure 4a). Further, the Cu plasticity can result in additional dislocations deposited at the shearing interface. Dislocations deposited at the Cu/Nb interface can lead to dislocation dissociation, which can affect the dislocation glide along the interface which can also contribute to the observed increase in the shear stress.

#### 3.1.2. Interface Plasticity in the RD Rectangular Micropillar Compression

In the case of the 63 nm Cu/Nb micropillar, the shear along the RD is theoretically prohibited due to extreme interfacial shear strength in this direction. Nevertheless, the interfacial shear along the RD is observed in Figure 3c,d. Later, because of high stress concentration built up at one of the corners of the micropillar, the shearing in the RD was resisted, thus activating the interface shearing along the TD, and hence the interface rotation observation. The nanolayer rotation towards the TD can be associated with the creation of stacking faults, which are perpendicular to the interface plane. The stacking fault is formed as a result of an extension of a partial dislocation, which originated from misfit dislocations with the Burgers vector perpendicular to the Cu/Nb ARB interface [14,39]. However, the rotation occurs at ≈155 MPa, which is below the theoretical shear stress of the stacking fault extension (≈300 MPa) [14,39]. This low critical stress can be attributed to pre-existing stacking faults in the Cu/Nb ARB rectangular micropillar. These pre-existing stacking faults allow a new stacking fault formation at low shear stress, as proposed by Zheng et al. [40]. The engineering shear stress vs. displacement curve for the RD micropillar shows that the shear stress is reduced to ≈100 MPa after the initiation of the layer rotational deformation (see Figure 3b). The major engineering stress drop in the RD micropillar can be associated with Cu layer failure, as shown in the inset in Figure 3c. Further deformation corresponds to a reduction of the contact area between the top and bottom part of the RD micropillar, which means that true shear stress is larger than the calculated ≈100 MPa engineering-resolved shear stress.

As shown in Figure 4, a comparison of the engineering-resolved shear stress versus indenter displacement for the TD and RD rectangular micropillars indicates that the RD micropillar exhibits yielding at a significantly lower stress (~155 MPa) compared to the TD micropillar (~300 MPa). As has been discussed more completely in the previous paragraph, it was due to high stress concentration built up at one of the corners of the micropillar as the shearing in the RD was resisted, thus activating the interface shearing along the TD, and hence the interface rotation observation. Hence, it should not be interpreted that shearing in the RD is easier than shearing in the TD. According to the molecular dynamic simulations by Wang et al. [41] and Demkowicz et al. [14], the interface shear strength along the RD is almost infinite for ARB Cu/Nb nanolaminate, though in the TD, interface shear strength would have a finite value. The RD micropillar was in effect plastically deformed at ~155 MPa via local plastic instability mechanisms other than the pure interface shearing in the rolling direction (RD) that the experiment was designed for.

These observations above were representative of a few rectangular micropillar compression experiments (4–5 micropillar samples) that we had conducted for each TD and RD micropillar compression in the present study. We were limited by the prohibitively high cost of the rectangular micropillar sample fabrication (using a focused ion beam (FIB)). Nevertheless, within this constraint, we observed the above phenomena (in TD as well as in RD micropillar compressions) consistently. All the TD micropillar compression experiments did not exhibit interfacial rotation—just straightforward interfacial shearing leading to failure (the shear yielding points may differ slightly—within a range of ±~100 MPa). Meanwhile, all the RD micropillar compressions ended up with interfacial rotation—again extent and actual rotating nanolayers may differ slightly from sample to sample, as well as in terms of the shear stress–displacement curves (qualitative shape, as shown in Figure 4b) and yield points (they differ within ±~150 MPa) in the present study. This could be associated with the pre-existing defects and other actual sample to sample variations, as has been described above. Interfacial rotation is clearly an important part of the overall interface-based plasticity mechanisms in the Cu/Nb ARB nanolaminate samples.

### 3.2. In Situ Cu/Nb ARB Microbeam Bending

In situ microbeam bending experiments were performed on Cu/Nb ARB nanolaminates (with layer thickness ~63 nm) to probe localized interfacial plasticity and fracture/failure resistance via crack growth/propagation through the thickness of the sample. It has been well documented that the layer thickness controls bulk and interfacial plasticity in bimetallic nanolaminates [22]. For ∼10<h<∼100 nm, where h is the distinct layer thickness, the motion of dislocation is typically governed by confined layer slip (CLS), which encompasses the motion of sole dislocation loops parallel to the interfaces within layers [24,42,43]. In the present work, as the layer thickness being studied is 63 nm, dislocation mechanisms may be expected to be governed by confined layer slip (CLS) which could induce enhanced ductility/toughness owing to limited plastic strain localization [44]. Apart from bulk localized plasticity of the constituent material layers, localized interfacial plasticity contributes to the overall plasticity of the nanolaminates. Interfacial plasticity which happens via nucleation and glide of interface dislocations parallel to the interface plane contributes to the overall plasticity [45]. In addition, in situ microbeam bending experiments were executed on the Cu/Nb ARB samples clamped along the TD—that is, to study the local interfacial plasticity mechanisms (near the notch) while the overall microbeam samples were stretched (pulled, in tension) along the transverse direction (TD) of the Cu/Nb ARB samples. From previous work [12,13], we may expect that interfacial shearing or sliding here would be much less inhibited by the dislocation configurations on the interfaces, compared to along the RD of the Cu/Nb ARB samples. This is related to the much higher interfacial shear strength along the RD (compared to TD), as predicted by Demkowicz et al. with molecular dynamics simulations [14].

#### 3.2.1. In Situ Microbeam Bending of Cu/Nb ARB Microbeam with No Offset Loading (Cu/Nb-NOL)

**Figure 5:**
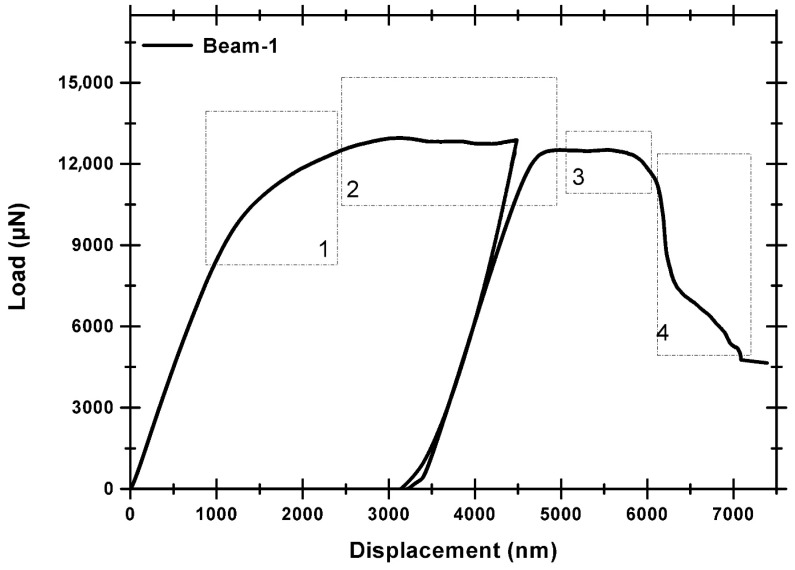
Load–displacement curve of ARB 63 nm Cu/Nb microbeam bending test with no offset loading (NOL) along TD: (1) the notch widens up until the instigation of crack, (2) additional notch widening with slight crack growth, (3) crack growth with notch widening, and (4) crack growth and formation of shear instabilities were suppressed due to the failure at one of the beam’s edges. No significant interfacial rotation was evident. Similar observations have been previously reported [15,48,49].

In situ microbeam bending of the Cu/Nb TD microbeam with no offset loading (Cu/Nb-NOL, Figure 5) exhibits notch widening, followed by crack initiation and propagation to final failure [12,13]. Along the TD, the Cu/Nb-NOL has low interfacial shear strength. The low interfacial shear stress along the TD can shear the interface in response to the stress fields generated by an adjoining lattice dislocation and could induce the dislocation in the interface [9,46]. These absorbed dislocations (irrespective of their sign) could extend their core inside the interface plane, which could result in localized interfacial plasticity. Also, these absorbed dislocations require re-nucleation in order to glide on the outward-bound slip system in the adjacent nanolayer. Therefore, the Cu/Nb-NOL TD microbeam provides stronger resistance to dislocation transmission and high localized interfacial plasticity and resistance to crack propagation [9,23].

The degree of notch widening for Cu/Nb-NOL was ~900% with respect to the initial notch width. Notch widening was calculated based on the original notch width (120 nm), and the final notch before the crack starts to propagate out of the notch corner across the thickness of the Cu/Nb-NOL. Typically, when the dislocations generated from the externally applied loading get deposited in the Cu/Nb interface, dislocation-mediated plasticity mechanisms get activated within the interface, which results in localized interfacial shear. Hence, the degree of notch widening discussed could be associated with interfacial shear strength/interfacial shear of the Cu/Nb nanolaminate.

In the case of Cu/Nb-NOL TD microbeam bending, high localized interfacial plasticity is typically observed [9,23]. Normally, in the instance of the Cu/Nb-NOL TD beam without any designed offset loading (in this work) or torque (in our previous work [47]), complex stress states do not develop. In general, the interface shear near the notch tip mostly follows the nominal stress state due to beam bending, i.e., layers of the Cu/Nb-NOL TD slide over each other near the tip of the notch, and this shearing action follows the general stress distribution caused by the bending of the beam. This is consistent with what we observed in this experiment (Cu/Nb-NOL). No significant interfacial rotation was observed near the location of the tip notch.

As the load is further increased, the dislocation starts to pile up at the corner of the notch to initiate crack. The propagation of crack through the thickness of the nanolaminate requires continuous generation and accumulation of dislocations. Nevertheless, dislocations may get absorbed, deposited, and annihilated in the lattice mismatched Cu/Nb-NOL TD interface, resulting in interfacial shear via dislocation-mediated localized plasticity mechanisms, thus hindering the propagation of the crack through the thickness of the microbeam. Subsequently, with an increase in the applied load, the crack would propagate, resulting in shear band formation and final failure. Nevertheless, in this particular case, crack propagation and subsequent shear band formation were hindered due to the failure of the microbeam at one of its fixed ends. Similar effects of low interfacial shear strength along the TD of Cu/Nb-NOL leading to localized interfacial plasticity and hindrances to the propagation of the crack, as well as the no significant interfacial rotation observation, have been previously reported [48,49] and established by means of finite element analysis [16].

As can be expected, during in situ microbeam bending, the interactions between dislocations generated due to the external load and interfaces could adjust the interface construction and orientation of slip systems across the interface, which could then potentially lead to some degree of interfacial rotation under certain circumstances. At substantially higher externally applied loads, these interfacial rotations do occur, which are essential for the alignment of slip systems for the flow of dislocations across the layers of Cu/Nb nanolaminate. Therefore, to a certain extent, the interfacial rotation is always possible in in situ microbeam bending with no offset loading or torque (Cu/Nb-NOL). Nevertheless, as is discussed subsequently in the next section (Section 3.1.2 Cu/Nb OL), the offset loading was purposely introduced in the instance of the Cu/Nb-OL microbeam (with offset loading) to generate complex stress states and hence subsequently leading to interfacial rotation.

#### 3.2.2. In Situ Microbeam Bending Experiments of Cu/Nb ARB Microbeam with Offset Loading (Cu/Nb-OL)

**Figure 6:**
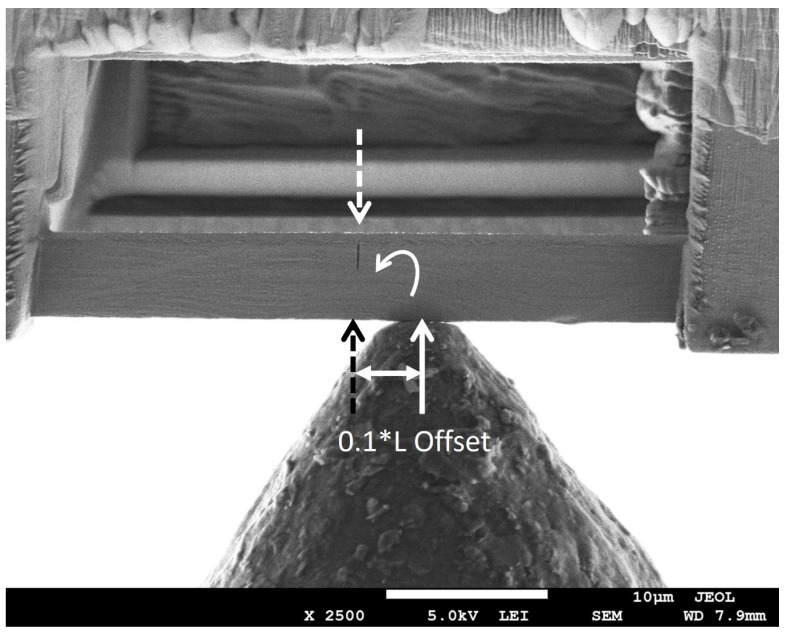
In situ microbeam bending tests were executed on pre-notched 63 nm Cu/Nb ARB clamped beam along the transverse direction (TD) with an offset loading. SEM image depicts a full beam with dimensions: Length = 40 µm, Width = 5.0 µm, and Thickness = 4.8 µm. The image depicts an offset loading applied to the beam with an Offset = 0.1 * Length (4 µm). The hypothetical dotted load lines are plotted to show that the offset loading results in a normal load at the notch tip (responsible for the bending of the beam) and a local bending moment restricting the flow of dislocations along the TD.

It has been noted in Section 3.2.1 that Cu/Nb-NOL showed appreciable localized interface plasticity along the TD, therefore, the aim here is to examine the significance of an offset loading on interfacial rotation, localized interface plasticity, and crack initiation and propagation in Cu/Nb nanolaminate (see Figure 6).

The introduction of offset loading induces the mixed mode loading in Cu/Nb-OL, which is otherwise similar to Cu/Nb-NOL in both in situ beam bending experimental parameters as well as the nominal beam geometry. Apart from the normal load component to induce the shear along the monolayers in the TD similar to Cu/Nb-NOL, a local bending moment is also introduced. The local bending moment was introduced to restrict the shear along the monolayers and thereby interfacial/bulk plasticity along the TD. The aim was to study if restriction of interfacial/bulk plasticity along the TD may induce interfacial shear along the RD and hence interfacial rotation.

**Figure 7:**
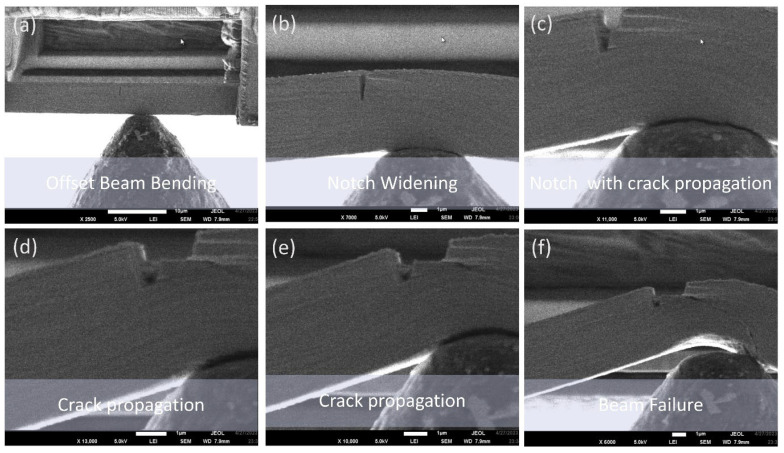
In situ microbeam bending test of 63 nm Cu/Nb TD microbeam with offset loading (OL): (**a**,**b**) the original notch widening, (**c**) notch widening trailed by crack instigation, (**d**–**f**) additional notch widening with crack evolution and spread through several layers from the notch tip, followed by eventual failure of the beam. Corresponding load–displacement curve is shown in Figure 8. Local interfacial rotation near the notch tip was evident (larger magnification image is provided in Figure 9).

**Figure 8:**
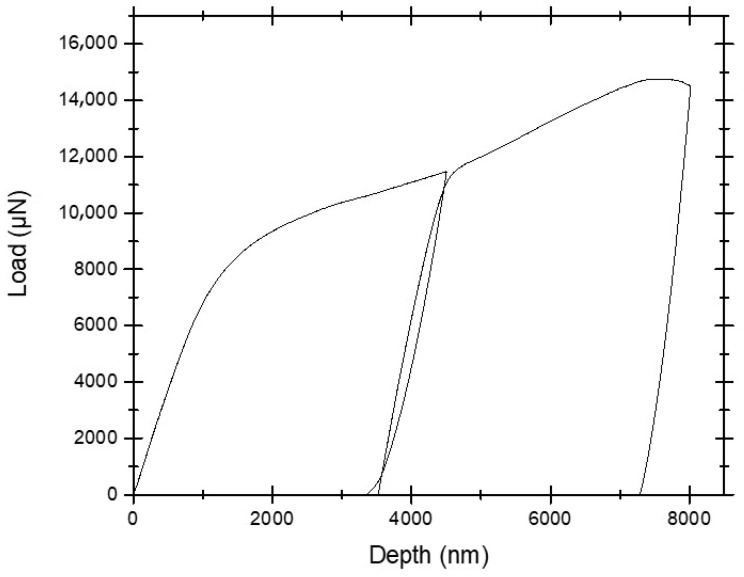
Load–displacement plot of 63 nm Cu/Nb TD microbeam (Cu/Nb-OL). The load rises with the applied displacement of the indenter tip due to the applied loading configuration shown in Figure 6, which constrains the interfacial shear along the TD but promotes the rotation along RD and subsequently shear band formation and eventual failure of the beam.

**Figure 9:**
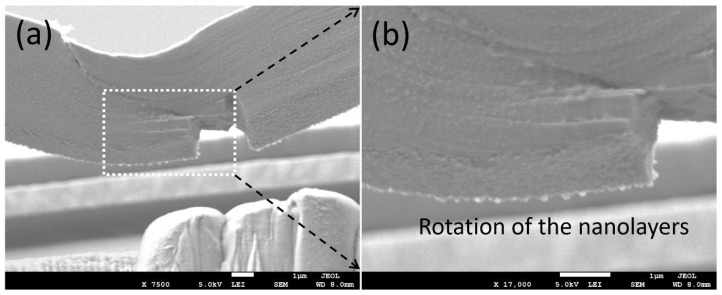
SEM images (**a**,**b**) display specifics of the area near the notch of Cu/Nb-OL. The variations of the surfaces around the notch (protrusion along RD) are attributed to the interfacial shear along the RD. Rotation of the nanolayers on the planes of the interfaces was evident, especially in (**b**). The horizontal direction here is TD, and the orthogonal direction into the image plane here is RD.

Cu/Nb-OL (Cu/Nb microbeam with offset loading) goes through similar regimes of notch widening and crack initiation, followed by its propagation and subsequent final failure (see Figure 5 and Figure 7). Nevertheless, the degree of the notch widening was rather low, around ~400%, compared to Cu/Nb-ARB-NOL (around ~900%). It is acknowledged that the motion of dislocations and thereby dislocation-mediated plasticity have a significant part in the deformation behavior of these Cu/Nb nanolaminates. Typically, dislocation transmission could occur across or along the interface of Cu/Nb nanolaminates, which could lead to the formation of shear band or interfacial shear, respectively, while in the case where dislocations do not transmit across or along the interface, interface tilting or rotation could occur [19]. In the present case, due to incorporation of the local bending moment at the notch tip, the interfacial shear was relatively more constrained along the TD (compared to Cu/Nb NOL), thus promoting interfacial shear along the RD (see Figure 8) and hence local interfacial rotation near the notch tip was evident (see Figure 9).

Also, the load–displacement plot of the 63 nm Cu/Nb-OL TD microbeam is plotted in Figure 8. The load rises with the applied displacement of the indenter tip into the sample mainly due to the applied loading configuration (see Figure 6). This applied loading configuration generates a combination of local bending moment and bending load which constrains the interfacial shear along the TD (constrains the flow of dislocation along the TD), while promoting the interfacial shear along the RD and hence local interfacial rotation (see Figure 9). Later, with an increase in the applied displacement, due to the local bending moment, the motion of dislocations is further constrained along the TD, resulting in dislocation pileup and subsequent crack propagation. The crack propagation consequently results in shear band formation and eventual failure of the beam. Such behavior was also observed during in situ TEM tensile tests of ARB 63 nm Cu/Nb nanolaminate by Liu et al. [50].

The observations above were representative of a few microbeam bending experiments (3–5 microbeams) that we had conducted for each Cu/Nb-NOL and Cu/Nb-OL in the current work. As discussed previously in Section 3.1, we were limited by the prohibitively high cost of the microbeam fabrication (using a focused ion beam (FIB)). However, within our limited number of experiments, we observed the above phenomena (in Cu/Nb-NOL as well as in Cu/Nb-OL) very consistently (only differed in the extent)—both in the notch widening and in the observation of interfacial rotation. We believe that the above experiments provide an important piece of the interfacial plasticity mechanisms in Cu/Nb ARB nanolaminates and thus key insights which could lead to the full understanding of the dislocation processes and their interaction with the interfaces both along the TD and RD under different loading configurations/conditions. The interfacial plasticity mechanism is an essential key for interface engineering and design for enhanced structural mechanical performance of nanomaterials in general and Cu/Nb nanolaminate-based mechanical components/structures specifically.

## 4. Discussion

Here, we discussed the experimental observations of interfacial plasticity mechanisms of Cu/Nb ARB nanolaminate samples particularly along the RD which is otherwise theoretically restricted due to high interfacial shear strength documented for the similar materials scheme [2,6,8,51,52,53], with our own work [15,16]. Typically, in Cu/Nb nanolaminates, owing to the lattice mismatch amongst copper and niobium, misfit dislocations are formed at the copper–niobium interface and reduce the overall strain energy. The interaction of these misfit dislocations with dislocations generated by externally applied loads governs the localized interfacial plasticity of the Cu/Nb nanolaminate involving the plasticity in the bulk nanolayers. When incoming dislocations due to externally applied load reach the interface, they can interact with the misfit dislocations through the creation, adsorption, or annihilation of dislocations. The occurrence and extent of these dislocation-mediated plasticity mechanisms depend on the specific characteristics of the misfit dislocations, such as their Burgers vector, line direction, and the crystallographic orientation of the sample involved. As the ARB Cu/Nb nanolaminates have distinct crystallographic orientation along the TD and RD, they depict distinct behavior of dislocation-mediated plasticity mechanisms along the TD and RD. The interfacial anisotropy of Cu/Nb nanolaminate has been extensively studied by means of experimental procedures [44,54,55,56] as well as computational modeling [8,14,41,57,58]. Molecular dynamic simulations by Demkowicz et al. [14] and Wang et al. [41] showed that, for ARB Cu/Nb nanolaminate, the interface shear strength is evidently infinite along the RD whereas it has a finite value along the TD. From here on, we stress our discussion on the importance of crystallographic orientation along the RD and its effect on the extent of interfacial rotation as observed in the present study.

It has been well documented in the literature from molecular dynamic simulations performed by Demkowicz et al. [14] and Wang et al. [41] that the interface shear strength is theoretically infinite to start interfacial shear along the RD [41,59]. Therefore, in situ rectangular micropillar compression experiments (with the Cu/Nb interfaces at ±45° from the loading axis) were performed along the RD and TD to directly probe the interface shear strength required to initiate dislocation-mediated plasticity along the RD and TD. These experiments serve as direct experimental verification of anisotropy in interface shear strength of the ARB Cu/Nb nanolaminate [14] explained in detail in Section 3.1. The in situ rectangular micropillar compression experiments clearly show that, although prohibited, interfacial shear could occur along the RD although accompanied by local plastic instabilities inducing other interface-mediated plasticity mechanisms, including interface rotation.

In the instance of microbeam bending, both Cu/Nb-NOL and Cu/Nb-OL exhibit notch widening along the TD, although the degree of the notch widening was higher in Cu/Nb-NOL (~900%) compared to Cu/Nb-OL (with offset loading), which was ~400% of the original notch width. The notch widening could be the result of both interfacial plasticity as well as bulk plasticity (within the nanolayers). Nevertheless, due to the extensive plastic deformation suffered by the constituent metal (especially Cu) in reduction from millimeter to nanometer scales during the ARB fabrication process, the available plasticity of the bulk material (within the nanolayers) is limited. Therefore, notch widening is primarily because of localized interfacial plasticity. This achievable interfacial shear strength along the TD (~1.2 GPa [14]) leads to interfacial shear in repsonse to the stress fields produced by an adjoining lattice dislocation under applied external load and induces the dislocation inside the interface [9,46]. Subsequently, the inward dislocation (regardless of the sign) gets absorbed and then spreads its core in the interior of the interface plane, and it will contribute to interfacial plasticity and thereby notch widening. To go across the interfaces, these dislocations would require re-nucleation to glide on the outward-bound slip system, which could happen only when extreme external stresses are imposed. Consequently, the ARB 63 nm Cu/Nb TD microbeam provides stronger resistance to slip transmission [9,23]. The higher notch widening for Cu/Nb-NOL is attributed to unrestricted interfacial plasticity along the TD compared to Cu/Nb-OL (with offset loading) where offset loading induced the local bending moment, restricting interfacial shearing along the TD.

Due to this restriction, we also observed interfacial rotation along the RD as has been described in Section 3.2.2 through in situ microbeam bending of the Cu/Nb ARB microbeam with offset loading (Cu/Nb-OL) (see Figure 9). It has been noted that, during this in situ microbeam bending of nanolaminate (Cu/Nb-OL), shear vectors were present in both the RD and TD, providing the possibility of layer rotational deformation in plane. In the case of Cu/Nb-OL, due to the constraint to the flow of dislocations along the TD because of offset loading, interfacial shear along the TD was restricted whereas interfacial shear along the RD was activated, resulting in interfacial rotation. The existence of interfacial rotation (from TD towards RD) was verified by analyzing the front and back surfaces of the notch after in situ beam bending testing (see Figure 9). In Figure 9, we observe layers have protruded along the RD, exhibiting non-uniformity across the nanolayers, particularly nearby the notch area. This deformation is ascribed to the interfacial rotation along the RD due to the constrained interfacial shear along the TD owing to the local bending moment intentionally induced in the Cu/Nb-OL (see Section 3.2.2).

**Figure 10:**
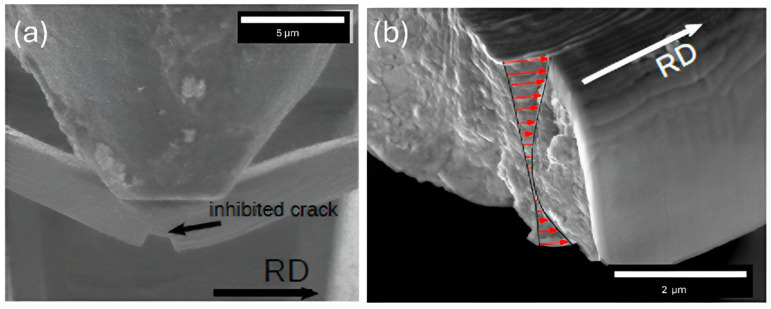
SEM images showing the microbeam bending experiment with the observation of interface rotation as earlier reported [47]: (**a**) the microbeam bending with offset loading setup and (**b**) the observation of interfacial rotation due to the mixed-mode loading. Due to offset loading, the microbeam is subjected to the mixture of Mode-I and Mode-II loading, which results in the interfacial rotation as shown in the figure [47]. Reproduced with permission from Elsevier.

It is worth noting here that interfacial rotation, a critical phenomenon observed in the present study, was also evident in our previous work. Specifically, we noted this behavior in our earlier publication, Anwarali et al. [47]. In that study, we briefly mentioned interface rotation observation but did not elaborate it as it was not the focus of that study. The present study allows us to discuss further and highlight this important observation. In Anwarali et al. [47], we demonstrated interfacial rotation during in situ microbeam bending experiments for unintended offset loading. Figure 10 pertaining to the work in Anwarali et al. [47] clearly illustrates the rotational deformation depicted via red arrows.

By presenting Figure 10 alongside Figure 7 and Figure 9 as have been shown earlier in the present manuscript, we provide additional visual proof of interface rotation occurring during in situ microbeam bending. This provides additional supporting evidence to our argument that this phenomenon is consistent and repeatable across different experiments with certain intended/unintended offset loading (offset loading introduces shear stresses at the crack tip, leading to a mixed-mode condition), validating the reliability of our findings. By emphasizing our previous work, we underline the continuity and progression of our research in this area, showcasing the consistency of our observations over several years and across multiple in situ microbeam bending experiments. This continuity underscores the depth of our work in understanding interfacial behavior, reinforcing the credibility and significance of our contributions to the field.

Also, offset loading in a clamped beam bending geometry significantly affects the mode of fracture and, consequently, the fracture toughness of a material [60]. This effect is crucial in understanding and predicting the behavior of materials under mixed-mode loading conditions, which often occur in practical engineering applications. Fracture toughness is a material’s capability to constrain crack spread under an applied load. It is quantified in terms of stress intensity factors, *K*_*I*_ for Mode-I (opening mode) and *K*_II_ for Mode-II (sliding mode) fractures. In Mode-I, the crack surfaces are pulled apart perpendicularly, while in Mode-II, the surfaces slide over each other in a parallel direction to the crack front. The fracture toughness for each mode, *K*_*I*C_ for Mode-I and *K*_IIC_ for Mode-II, depends on the stress state nearby the crack tip, which can be changed by changing the loading conditions.

**Figure 11:**
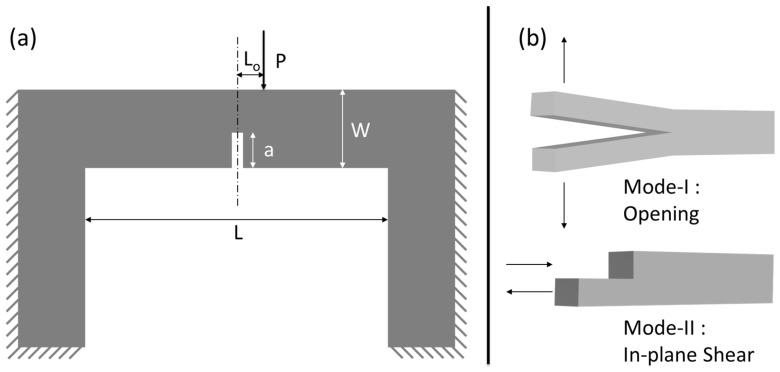
Schematic illustrations of the in situ clamped microbeam bending setup with offset loading: (**a**) in situ clamped microbeam bending having a centered notch with offset loading configuration, and (**b**) schematic illustrations of Mode-I (opening) and Mode-II (in-plane shear).

In situ clamped microbeam bending is being used for evaluating fracture toughness, particularly because it provides a stable configuration for crack growth/propagation. In this geometry, the microbeam is clamped at both ends, and a load is applied at a point between the clamps (see Figure 11a). The position of the load relative to the notch (offset distance, *L*_o_) determines the mixity of the loading mode—whether it induces Mode-I, Mode-II, or both (mixed mode) (see Figure 11b). When the load is applied directly above the crack (i.e., *L*_o_ = 0), the loading condition is symmetric with respect to the crack line. This configuration induces pure Mode-I loading, where the crack faces are pulled apart perpendicularly to the crack front. The stress intensity factor $K_{I}$ dominates, and the material’s fracture toughness is governed primarily by $K_{Ic}$, the Mode-I fracture toughness.

(1) $$K_{I}=\frac{PL}{BW^{2}}\sqrt{\pi aY_{I}}\left(\frac{a}{W},\frac{L_{0}}{L}\right)$$

(2) $$K_{II}=\frac{PL}{BW^{2}}\sqrt{\pi aY_{II}}\left(\frac{a}{W},\frac{L_{0}}{L}\right)$$

where *P*, *W*, *B*, *L*, *L*_0_ and *a* are external applied load, width of the microbeam, thickness of the microbeam, length of the microbeam, offset distance, and initial crack length, respectively. $Y_{I}$ and $Y_{II}$ are mode I and mode II normalized geometric stress intensity factors, respectively, a function of relative crack length (*a*/*W*) and relative offset (*L*_0_/*L*) [60].

As the load application point is moved away from the notch line (i.e., *L*_o_ > 0), an asymmetry is introduced in the loading condition. The offset loading introduces shear stresses at the crack tip, leading to a mixed-mode condition. This condition combines Mode-I and Mode-II loading together, with the proportion of Mode-II increasing as *L*_o_ increases. The stress intensity factors $K_{I}$ and $K_{II}$ now both contribute to the crack propagation. The fracture toughness is no longer governed solely by $K_{Ic}$ but $K_{IIc}$, the Mode-II fracture toughness, depending on the degree of mode mixity.

Finite element method simulations [60] were employed to quantify the effect of offset loading on the stress intensity factors $K_{I}$ and $K_{II}$ (Figures 11 and 2 (Chapter 4: Clamped Beam Bending for Mixed Mode Fracture Toughness Measurements) [60] and Equations (1) and (2)). With increasing *L*_o_, the normalized $K_{I}$ decreases, indicating a reduction in Mode-I contribution. Simultaneously, the normalized $K_{II}$ increases, indicating an increasing Mode-II contribution. However, after a certain point $K_{II}$ begins to decrease again, signifying the complex interplay between the two modes. The fracture toughness measured under mixed-mode conditions differs from that measured under pure Mode-I or Mode-II conditions. The toughness is influenced by the ratio $K_{II}$/$K_{I}$, which varies with *L*_o_. For materials that exhibit higher resistance to Mode-II (shear) than Mode-I (tensile) loading, the fracture toughness increases with increasing *L*_o_. However, the exact relationship depends on the material’s intrinsic properties and the specific geometry of the test specimen.

Similarly, in the present study, due to offset loading, the Cu/Nb ARB microbeam experiences a mixture of Mode-I and Mode-II loading (Figure 12 in Reference [60]). For the current fixed beam bending with offset loading configuration, *L*_o_/L = 0.1, L/W = 8, and a/W = 0.3 replicate the scenario in Figure 2b (Chapter 4: Clamped Beam Bending for Mixed Mode Fracture Toughness Measurements) [60], which clearly shows that $K_{I}$ is reducing whereas $K_{II}$ is increasing, depicting a mixed-mode configuration. Due to the presence of Mode-I, the crack surfaces are pulled apart perpendicularly, resulting in interfacial sliding and subsequent notch widening, while due to the presence of Mode-II, the layers of Cu/Nb ARB nanolaminate slide over each other in a parallel direction to the crack front, resulting in the interfacial rotation as visible in Figure 7, Figure 9, and Figure 10.

Comparing the two small-scale mechanical testing experiments of the Cu/Nb ARB nanolaminate samples, in situ rectangular micropillar compression offers more straightforward experimental evidence of interfacial rotation in Cu/Nb ARB nanolaminates. In micropillar compression experiments, there was only the nominal shear stress (as the driving force of deformation) in either the RD or TD. The loading allows the deformation in the Cu/Nb ARB interfaces either in the RD or TD, practically unconstrained by any other macroscale (i.e., non-material) conditions. Despite nominally only shear stress in the rolling direction (RD), our RD micropillar compression experiments exhibit clear evidence of interfacial shearing also in the TD, hence the observation of the interfacial rotation (from RD towards TD) of the Cu/Nb nanolayers. The interfacial rotation was also evident in the in situ microbeam bending with offset loading (Cu/Nb-OL), but the microbeam bending mode here could lead to relatively more constraints (compared to a rectangular micropillar) and thus much more complex stress states, especially near the notch tip, as has been discussed in Section 3.1.2.

Nevertheless, the experimental evidence in this present study has clearly shown interfacial rotation of Cu/Nb nanolayers in the Cu/Nb ARB nanolaminate samples, despite no rotational driving force from the loading mode (especially in the micropillar compression). Our aim in the present manuscript is to report this basic phenomenon. Our experimental results in the present study on localized interfacial plasticity along the TD and RD in Cu/Nb nanolaminates have revealed the complex interplay between dislocation-mediated localized plasticity, interfacial structure, and applied loading configurations/conditions. Interfacial rotation is certainly an interesting phenomenon, but more importantly, without the full understanding of interfacial rotation and interface-mediated plasticity mechanisms more generally, we could not carry out interface engineering and design for enhanced structural mechanical performance of nanomaterials in general and Cu/Nb nanolaminate-based mechanical components/structures specifically. We believe the present study provides sufficient justification for further, more in-depth, and quantitative studies of the interface rotation mechanism in Cu/Nb ARB nanolaminate materials.

## 5. Conclusions

In situ microbeam bending as well as in situ micropillar compression experiments of Cu/Nb nanolaminates have revealed evidence regarding the dislocation-mediated localized plasticity along the RD and TD and the possible impact on the design/production of Cu/Nb-nanolaminate-based components/structures subjected to one or a combination of compression, torsional, and bending loading configurations. Interfacial shear and rotation were observed along both the RD and TD during in situ rectangular pillar compression, despite theoretical predictions suggesting that shear should be inhibited along the RD. Offset in situ microbeam bending along the TD introduced a local bending moment which restricted interfacial shear along the TD but generated interfacial shear/rotation along the RD. These outcomes of the study could be very significant for advanced structural applications, as they offer understandings of applied loading configurations/conditions and their effect on the interfacial shear/rotation along the TD and RD for anisotropic Cu/Nb nanolaminate. The authors think that information regarding interfacial sliding/rotation under diverse loading configurations could help researchers in scheming workable design diagrams for a given material system, which could motivate the design/fabrication of novel strong/tough assemblies sustainable under complex loading configurations/conditions for evolving functionalities, like stretchable bimetallic conductors for innovative wearable devices.

## Data Availability

The original contributions presented in this study are included in the article. Further inquiries can be directed to the corresponding author(s).

## References

[1] Holleck H., Schier V. (1995). Multilayer PVD coatings for wear protection. Surf. Coat. Technol..

[2] Beyerlein I.J., Mara N.A., Carpenter J.S., Nizolek T., Mook W.M., Wynn T.A., McCabe R.J., Mayeur J.R., Kang K., Zheng S. (2013). Interface-driven microstructure development and ultra high strength of bulk nanostructured Cu-Nb multilayers fabricated by severe plastic deformation. J. Mater. Res..

[3] Hoagland R.G., Mitchell T.E., Hirth J.P., Kung H. (2002). On the strengthening effects of interfaces in multilayer fee metallic composites. Philos. Mag. A.

[4] Han W., Cerreta E., Mara N., Beyerlein I., Carpenter J., Zheng S., Trujillo C., Dickerson P., Misra A. (2014). Deformation and failure of shocked bulk Cu–Nb nanolaminates. Acta Mater..

[5] Misra A., Demkowicz M.J., Zhang X., Hoagland R.G. (2007). The radiation damage tolerance of ultra-high strength nanolayered composites. JOM.

[6] Demkowicz M., Wang Y., Hoagland R., Anderoglu O. (2007). Mechanisms of He escape during implantation in CuNb multilayer composites. Nucl. Instrum. Methods Phys. Res. Sect. B Beam Interact. Mater. Atoms.

[7] Lim S., Rollett A. (2009). Length scale effects on recrystallization and texture evolution in Cu layers of a roll-bonded Cu–Nb composite. Mater. Sci. Eng. A.

[8] Beyerlein I., Demkowicz M., Misra A., Uberuaga B. (2015). Defect-interface interactions. Prog. Mater. Sci..

[9] Wang J., Misra A. (2011). An overview of interface-dominated deformation mechanisms in metallic multilayers. Curr. Opin. Solid State Mater. Sci..

[10] Shao S., Medyanik S.N. (2010). Dislocation–interface interaction in nanoscale fcc metallic bilayers. Mech. Res. Commun..

[11] Zhang R., Germann T., Wang J., Liu X.-Y., Beyerlein I. (2013). Role of interface structure on the plastic response of Cu/Nb nanolaminates under shock compression: Non-equilibrium molecular dynamics simulations. Scr. Mater..

[12] Radchenko I., Zhu W., Qing L., Navarro E., Sahay R., Lee P.S., Chen K. (2022). Interface Rotation in Cu/Nb Accumulative Roll Bonded (ARB) Nanolaminates. SSRN Electron. J..

[13] Sahay R., Budiman A.S., Aziz I., Navarro E., Escoubas S., Cornelius T.W., Gunawan F.E., Harito C., Lee P.S., Thomas O. (2022). Crystallographic Anisotropy Dependence of Interfacial Sliding Phenomenon in a Cu(16)/Nb(16) ARB (Accumulated Rolling Bonding) Nanolaminate. Nanomaterials.

[14] Demkowicz M., Thilly L. (2011). Structure, shear resistance and interaction with point defects of interfaces in Cu–Nb nanocomposites synthesized by severe plastic deformation. Acta Mater..

[15] Ali H.P.A., Budiman A. (2019). Advances in In situ microfracture experimentation techniques: A case of nanoscale metal–metal multilayered materials. J. Mater. Res..

[16] Radchenko I., Anwarali H.P., Tippabhotla S.K., Budiman A.S. (2018). Effects of interface shear strength during failure of sem-icoherent metal–metal nanolaminates: An example of accumulative roll-bonded Cu/Nb. Acta Mater..

[17] Ali H.P.A., Radchenko I., Li N., Budiman A. (2018). The roles of interfaces and other microstructural features in Cu/Nb nanolayers as revealed by in situ beam bending experiments inside an scanning electron microscope (SEM). Mater. Sci. Eng. A.

[18] Ali H.P.A., Radchenko I., Li N., Budiman A. (2019). Effect of multilayer interface through in situ fracture of Cu/Nb and Al/Nb metallic multilayers. J. Mater. Res..

[19] Zheng S., Wang J., Carpenter J., Mook W., Dickerson P., Mara N., Beyerlein I. (2014). Plastic instability mechanisms in bimetallic nanolayered composites. Acta Mater..

[20] Saito Y., Utsunomiya H., Tsuji N., Sakai T. (1999). Novel ultra-high straining process for bulk materials—Development of the accumulative roll-bonding (ARB) process. Acta Mater..

[21] Wang J., Hoagland R., Liu X., Misra A. (2011). The influence of interface shear strength on the glide dislocation–interface interactions. Acta Mater..

[22] Wang J., Zhou Q., Shao S., Misra A. (2017). Strength and plasticity of nanolaminated materials. Mater. Res. Lett..

[23] Wang J., Misra A., Hoagland R., Hirth J. (2012). Slip transmission across fcc/bcc interfaces with varying interface shear strengths. Acta Mater..

[24] Misra A., Hirth J.P., Kung H. (2002). Single-dislocation-based strengthening mechanisms in nanoscale metallic multilayers. Philos. Mag. A.

[25] Zheng S., Beyerlein I.J., Carpenter J.S., Kang K., Wang J., Han W., Mara N.A. (2013). High-strength and thermally stable bulk nanolayered composites due to twin-induced interfaces. Nat. Commun..

[26] Nix W.D., Gao H. (1998). Indentation size effects in crystalline materials: A law for strain gradient plasticity. J. Mech. Phys. Solids.

[27] Wu K., Zhang J., Zhang P., Wang Y., Liu G., Zhang G., Sun J. (2014). Fracture behavior and adhesion energy of nanostructured Cu/Mo multilayer films. Mater. Sci. Eng. A.

[28] Greer J.R., Oliver W.C., Nix W.D. (2005). Size dependence of mechanical properties of gold at the micron scale in the absence of strain gradients. Acta Mater..

[29] Greer J.R., De Hosson J.T.M. (2011). Plasticity in small-sized metallic systems: Intrinsic versus extrinsic size effect. Prog. Mater. Sci..

[30] Mara N.A., Bhattacharyya D., Dickerson P., Hoagland R.G., Misra A. (2008). Deformability of ultrahigh strength 5 nm Cu/Nb nanolayered composites. Appl. Phys. Lett..

[31] Zhang H., Schuster B., Wei Q., Ramesh K. (2006). The design of accurate micro-compression experiments. Scr. Mater..

[32] Li N., Mara N., Wang J., Dickerson P., Huang J., Misra A. (2012). Ex situ and in situ measurements of the shear strength of interfaces in metallic multilayers. Scr. Mater..

[33] Jaya B.N., Kirchlechner C., Dehm G. (2015). Can microscale fracture tests provide reliable fracture toughness values? A case study in silicon. J. Mater. Res..

[34] Mayer C., Yang L., Singh S., Llorca J., Materialia J.L.-A., Shen Y.L., Chawla N. (2016). Anisotropy, size, and aspect ratio effects on micropillar compression of AlSiC nanolaminate composites. Acta Mater..

[35] Zeng Z., Xiao Y., Wheeler J.M., Tan J.-C. (2023). In situ micropillar compression of an anisotropic metal-organic framework single crystal. Commun. Chem..

[36] Maeder X., Mook W., Niederberger C., Michler J. (2011). Quantitative stress/strain mapping during micropillar compression. Philos. Mag..

[37] Dimiduk D.M., Woodward C., LeSar R., Uchic M.D. (2006). Scale-free intermittent flow in crystal plasticity. Science.

[38] Weygand D., Poignant M., Gumbsch P., Kraft O. (2008). Three-dimensional dislocation dynamics simulation of the influence of sample size on the stress–strain behavior of fcc single-crystalline pillars. Mater. Sci. Eng. A.

[39] Zhou J., Averback R., Bellon P. (2014). Stability and amorphization of Cu–Nb interfaces during severe plastic deformation: Molecular dynamics simulations of simple shear. Acta Mater..

[40] Zheng S., Beyerlein I., Wang J., Carpenter J., Han W., Mara N. (2012). Deformation twinning mechanisms from bimetal interfaces as revealed by in situ straining in the TEM. Acta Mater..

[41] Wang J., Kang K., Zhang R.F., Zheng S.J., Beyerlein I.J., Mara N.A. (2012). Structure and property of interfaces in ARB Cu/Nb laminated composites. JOM.

[42] Anderson P.M., Bingert J.F., Misra A., Hirth J.P. (2003). Rolling textures in nanoscale Cu/Nb multilayers. Acta Mater..

[43] Anderson P., Foecke T., Hazzledine P. (1999). Dislocation-based deformation mechanisms in metallic nanolaminates. MRS Bull..

[44] Nizolek T., Beyerlein I.J., Mara N.A., Avallone J.T., Pollock T.M. (2016). Tensile behavior and flow stress anisotropy of accumulative roll bonded Cu-Nb nanolaminates. Appl. Phys. Lett..

[45] Wang J., Hoagland R., Hirth J., Misra A. (2008). Atomistic simulations of the shear strength and sliding mechanisms of copper–niobium interfaces. Acta Mater..

[46] Wang J., Hoagland R.G., Hirth J.P., Misra A. (2008). Atomistic modeling of the interaction of glide dislocations with “weak” interfaces. Acta Mater..

[47] Budiman A.S., Sahay R., Ali H.P.A., Tippabhotla S.K., Radchenko I., Raghavan N. (2021). Interface-mediated plasticity and fracture in nanoscale Cu/Nb multilayers as revealed by in situ clamped microbeam bending. Mater. Sci. Eng. A.

[48] Pilkey W.D., Pilkey D.F. (2008). Peterson’s Stress Concentration Factors.

[49] Li Y., Zhou Q., Zhang S., Huang P., Xu K., Wang F., Lu T. (2018). On the role of weak interface in crack blunting process in nanoscale layered composites. Appl. Surf. Sci..

[50] Liu Z., Monclús M., Yang L., Castillo-Rodríguez M., Molina-Aldareguía J., Llorca J. (2018). Tensile deformation and fracture mechanisms of Cu/Nb nanolaminates studied by in situ TEM mechanical tests. Extrem. Mech. Lett..

[51] Carpenter J.S., McCabe R.J., Zheng S.J., Wynn T.A., Mara N.A., Beyerlein I.J. (2014). Processing parameter influence on texture and microstructural evolution in Cu-Nb multilayer composites fabricated via accumulative roll bonding. Metall. Mater. Trans. A.

[52] Hattar K., Misra A., Dosanjh M.R.F., Dickerson P., Robertson I.M., Hoagland R.G. (2012). Direct observation of crack propagation in copper–niobium multilayers. J. Eng. Mater. Technol..

[53] Beyerlein I., Mayeur J., McCabe R., Zheng S., Carpenter J., Mara N. (2014). Influence of slip and twinning on the crystallographic stability of bimetal interfaces in nanocomposites under deformation. Acta Mater..

[54] Demkowicz M.J., Beyerlein I.J. (2020). The effects of nanoscale confinement on the behavior of metal laminates. Scr. Mater..

[55] Chen T., Yuan R., Beyerlein I.J., Zhou C. (2020). Predicting the size scaling in strength of nanolayered materials by a discrete slip crystal plasticity model. Int. J. Plast..

[56] Dong S., Chen T., Huang S., Li N., Zhou C. (2020). Thickness-dependent shear localization in Cu/Nb metallic nanolayered composites. Scr. Mater..

[57] Budiman A.S., Han S.-M., Li N., Wei Q.-M., Dickerson P., Tamura N., Kunz M., Misra A. (2012). Plasticity in the nanoscale Cu/Nb single-crystal multilayers as revealed by synchrotron Laue x-ray microdiffraction. J. Mater. Res..

[58] Misra A., Demkowicz M.J., Wang J., Hoagland R.G. (2008). The multiscale modeling of plastic deformation in metallic nanolayered composites. JOM.

[59] Mara N.A., Bhattacharyya D., Hirth J.P., Dickerson P., Misra A. (2010). Mechanism for shear banding in nanolayered composites. Appl. Phys. Lett..

[60] Jonnalagadda K., Alankar A., Balila N.J., Bhandakkar T. (2022). Advances in Structural Integrity: Structural Integrity over Multiple Length Scales.

